# Wishbone Ingestion Leading to a Diagnosis of Occult Sigmoid Adenocarcinoma

**DOI:** 10.7759/cureus.111820

**Published:** 2026-06-30

**Authors:** Natalie Rosseau, Hannah Zuercher, Ahmed Al Qady, Anand Gupte, John Lieb

**Affiliations:** 1 Division of Internal Medicine, University of Florida College of Medicine, Gainesville, USA; 2 Division of Gastroenterology, Hepatology, and Nutrition, University of Florida College of Medicine, Gainesville, USA

**Keywords:** case report, colonic perforation, colorectal cancer, ingested foreign body, wishbone

## Abstract

Foreign body ingestion can lead to life-threatening complications, including colonic perforation, especially with sharp-pointed objects. We detail an unusual case of a male patient in his seventies who presented with left lower quadrant abdominal pain and diarrhea, found to be secondary to a sharp foreign body ingestion, a wishbone. This finding led to a diagnosis of sigmoid adenocarcinoma, followed by colonic perforation weeks after an unsuccessful endoscopic removal attempt and prior to planned surgical intervention. He underwent exploratory laparotomy with wishbone removal, sigmoid colectomy, and diverting loop ileostomy. He was discharged to a skilled nursing facility for postoperative recovery with vacuum-assisted midline incision therapy. Our case is one of the few to detail a malignant mass causing colonic foreign body impaction with attempted endoscopic intervention, highlighting the intricacies of managing foreign body ingestion with endoscopic versus surgical intervention.

## Introduction

Foreign body ingestion is most commonly observed in pediatric patients, individuals with psychiatric disorders, and those with gastrointestinal (GI) motility abnormalities [1]. As most foreign bodies pass spontaneously through the GI tract, the risk of mortality remains low. However, perforation, obstruction, and peritonitis may still arise, with heightened risk in those with a history of GI surgery, congenital gut malformations, GI malignancies, or sharp object ingestion [1-2]. 

Ingestion of sharp objects poses significant diagnostic and therapeutic difficulties, as they may be challenging to identify radiographically, and retrieval carries an increased risk of injury, with complications occurring in up to 35% of cases [1-3]. Therefore, the American Society for Gastrointestinal Endoscopy (ASGE) recommends emergent removal of sharp foreign bodies [4]. Endoscopic removal is the first-line approach for managing ingested sharp objects, utilizing techniques such as a polypectomy snare, retrieval net, or retrieval forceps [3]. If these endoscopic methods fail, surgical intervention should then be considered [4]. The management of a sharp foreign body impacted in a suspicious mass or known malignant stricture is more complex and therefore less clear.

Here, we present a case of a sharp foreign body ingestion, a wishbone, leading to an incidental finding of sigmoid adenocarcinoma, complicated by colonic perforation weeks after an unsuccessful endoscopic removal attempt and prior to planned surgical intervention. This article was previously presented as a poster at the 2025 American College of Gastroenterology Annual Scientific Meeting on October 28, 2025.

## Case presentation

**Figure 1:**
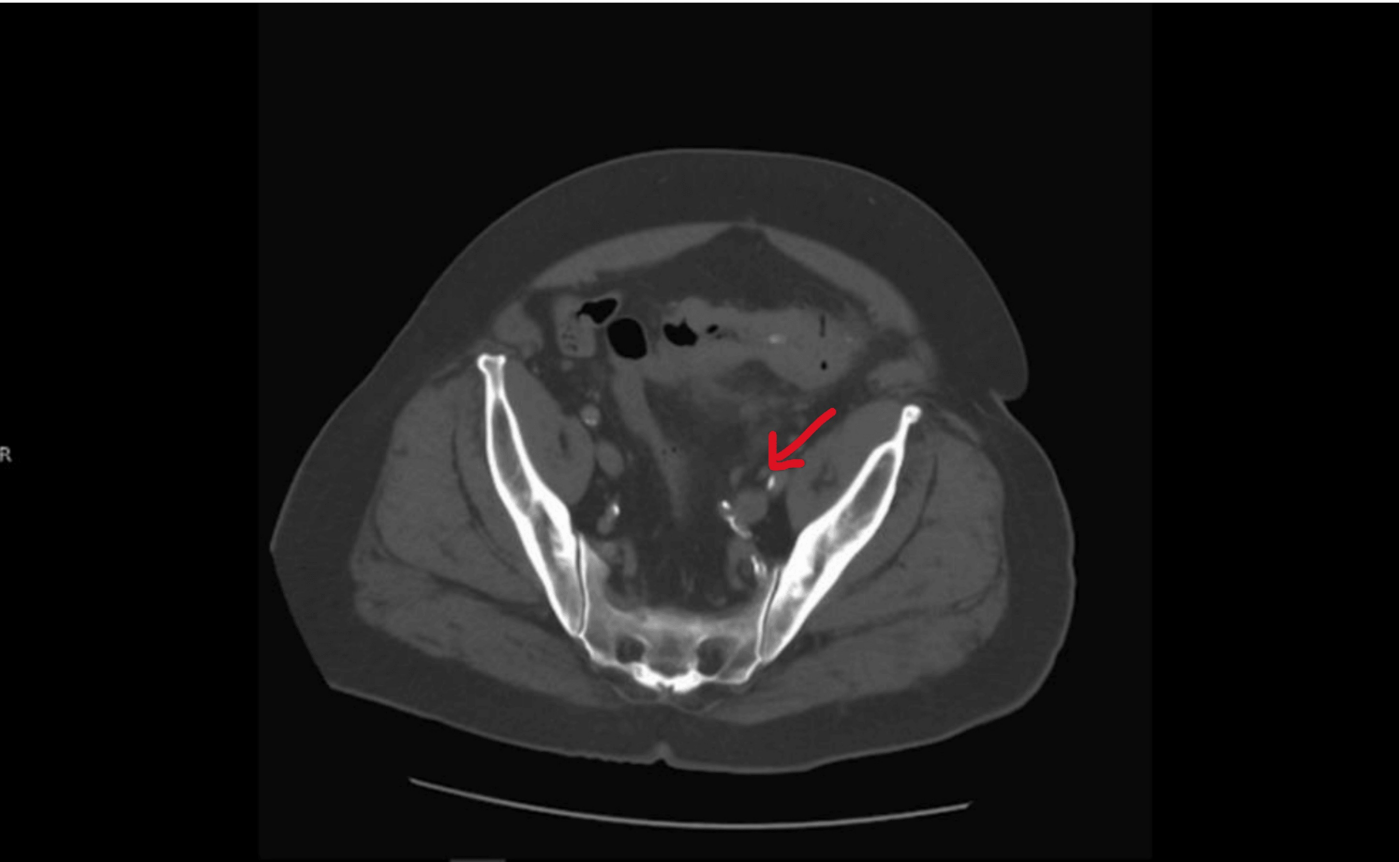
The ingested wishbone visualized radiographically in the transverse plane with intravenous contrast at the level of the sigmoid colon, demonstrating a proximal thickened bowel segment.

A male in his seventies with a past medical history of type 2 diabetes mellitus, coronary artery disease with four-vessel coronary artery bypass graft, infrarenal abdominal aortic aneurysm, hypertension, hyperlipidemia, and obesity presented to the emergency department of the Malcolm Randall Department of Veterans Affairs Medical Center at Gainesville, FL, USA, with a two-week history of left lower quadrant abdominal pain and ten episodes of diarrhea per day for two days. He had never received a colonoscopy. He was afebrile and hemodynamically stable upon presentation. Initial laboratory analysis was notable for hemoglobin of 12.7 g/dL (normal range: 13.9-18.0 g/dL) and white blood cell count of 9 x 10³ k/cm (normal range: 4.6-10.8 k/cm). Computed tomography (CT) of the abdomen and pelvis with contrast noted circumferential mural thickening of a short segment of sigmoid colon with an ingested wishbone impacted at the proximal thickened bowel segment (Figure 1).

**Figure 2:**
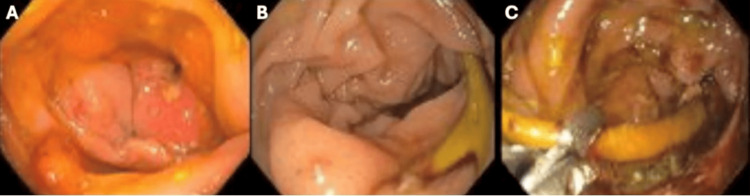
Ingested wishbone visualized endoscopically A. Endoscopic image of a 4-5 centimeter friable, partially-obstructing circumferential mass in the sigmoid colon, causing luminal narrowing. B. Endoscopic image of the ingested wishbone impacted proximal to the mass. C. Endoscopic image of attempted wishbone removal using retrieval forceps.

Given the atypical foreign body impaction location, colonic malignancy was suspected. The orientation of the wishbone and the presence of a mass were confirmed endoscopically. Colonoscopy demonstrated a sigmoid colonic friable, partially obstructing circumferential mass measuring 4-5 centimeters in length, causing luminal narrowing (Figure 2A). Proximal to the mass was an impacted wishbone (Figure 2B). There was no evidence of mucosal injury. Attempts were made to disimpact the wishbone from the mass using grasper forceps. Some resistance was encountered, and endoscopic attempts to remove the wishbone were aborted. These removal attempts were unsuccessful due to the wishbone's Y-shape abutting the colonic mass (Figure 2C).

Carcinoembryonic antigen (CEA) level was 14.1 ng/mL (normal range: 0-5 ng/mL). The surgical team was reconsulted after endoscopic removal attempts failed. Given that there was no hemodynamic compromise or evidence of perforation, outpatient surgical removal was recommended. The patient was discharged with follow-up. 

**Figure 3:**
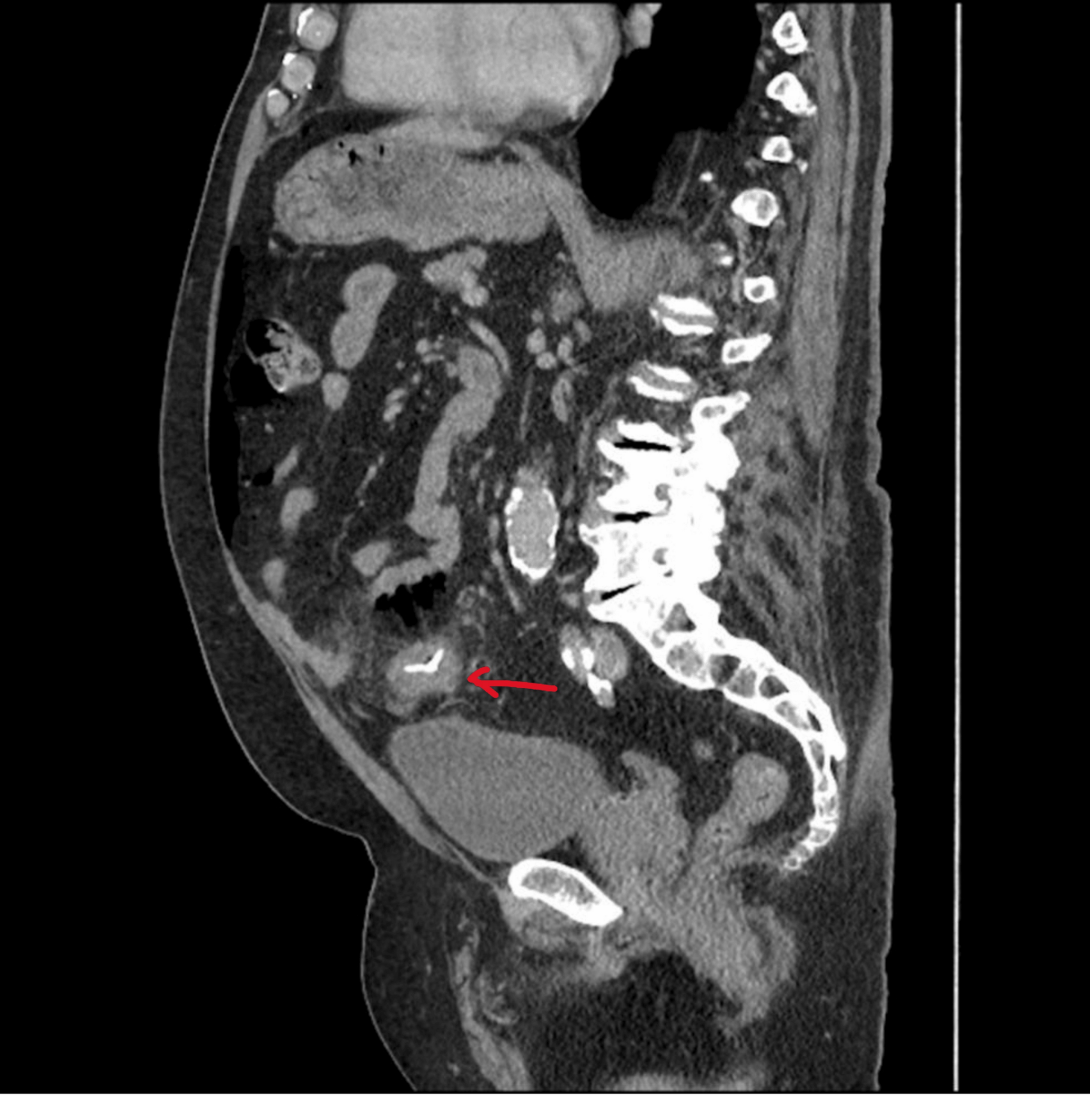
Redemonstration of the ingested wishbone on sagittal contrast-enhanced radiographic imaging at the level of the sigmoid colon, with a proximally thickened bowel segment, new adjacent free air, and a moderate volume of free air throughout the abdomen, concerning for bowel perforation.

He re-presented two weeks later with worsening abdominal pain and distension, fever, and focal peritoneal signs. A CT of the abdomen and pelvis redemonstrated wishbone impaction, with adjacent free air concerning for sigmoid perforation (Figure 3).

He underwent exploratory laparotomy with wishbone removal, sigmoid colectomy, and diverting loop ileostomy. Pathology noted a 5.7-centimeter, moderately differentiated sigmoid colon stage tumor 3 lymph node 0 (T3N0) adenocarcinoma, with the margins free of tumor. The 26 examined lymph nodes were negative for metastatic carcinoma. He was discharged to a skilled nursing facility for postoperative recovery with vacuum-assisted midline incision therapy.

## Discussion

For sharp foreign bodies that have migrated beyond the ligament of Treitz, a minimally invasive approach via deep enteroscopy or colonoscopy can be attempted to remove the object to avoid distal perforation and surgical intervention. Devices to facilitate sharp foreign body removal via endoscopy include retrieval forceps, baskets, and polypectomy snares. While these tools may be similar to those used in antegrade endoscopy and sharp foreign body removals, the proposed technique for removal differs in the retrograde approach. In contrast to trailing the sharp point as is customary for an antegrade approach, approaches via colonoscopy should consider covering the pointed end prior to drawing it into the scope to avoid mucosal injury [5]. While endoscopic removal was unsuccessful in our case, it is important to note that the obstruction was caused by malignancy, making traditional extraction techniques more difficult. 

Although foreign body ingestion is not unusual, our case highlights several points worth a clinician's consideration. There have been limited reports of swallowed bone ingestion published within this decade [6]. While ingested foreign bodies are often encountered in gastroenterology, colonic impaction is less common. Interestingly, our patient did not recall ingesting a wishbone. While it is well documented that patients often do not recall the event during which they ingested a foreign body, this patient did not have typical risk factors for ingestion, including young or advanced age, usage of dentures or missing dentition, a recent history of alcohol abuse, psychiatric diagnoses, or neurocognitive comorbidities [6-9]. Additionally, our patient initially did not present with bowel perforation. Most documented foreign body ingestions of animal bones present with bowel perforation, requiring surgical intervention as treatment [7-9]. In our case, the wishbone was unable to be endoscopically removed due to a malignant stricture in the sigmoid colon, and later presented with bowel perforation prior to scheduled surgical removal. 

The decision to pursue endoscopic intervention as the initial treatment of this foreign body ingestion is also worth discussing. While general surgery and gastroenterology were both consulted in the emergency department, general surgery deferred to the gastroenterology team for endoscopic management of the foreign body. The surgical team was made aware that the foreign body was unable to be removed endoscopically and elected to pursue outpatient management due to the patient’s clinical stability. While controversial, this decision to pursue watchful waiting was potentially justifiable based on reports that found that most ingested foreign bodies pass spontaneously and uneventfully. In retrospect, failed endoscopic intervention should have led to a more robust discussion with surgical colleagues about the risk of immediate surgery versus admission for watchful waiting versus staged surgical intervention as an outpatient [10, 11]. This case highlights the importance of interdisciplinary dialogue with consulting services, particularly if these recommendations could pose a potential danger to the patient.

The literature does not clearly outline when surgical intervention should be favored over endoscopic foreign body removal in the setting of a malignant stricture in the lower GI tract. For foreign bodies in the upper GI tract, Lee et al. found that most foreign bodies in their study could be successfully removed by endoscopic techniques, with age > 70 years, foreign bodies in the upper esophagus, maximum diameter > 30 mm, and impaction time > 40 hours cited as significant risk factors for predicting conversion to surgery [12]. In their study of foreign body ingestion throughout the GI tract, Gallagher et al. noted that foreign body length was more associated with the need for operative intervention than the shape, finding that pointed objects were actually associated with a lower risk for needing surgery [13]. Among cases of lower GI tract foreign bodies, Wu et al. identified foreign body-intestinal canal angle >51.25° and free peritoneal gas as computed tomography imaging predictors for surgical intervention [14]. This case highlights the need for additional studies that would support the development of guidelines to support clinical management of patients with foreign bodies impacted in malignant strictures in the lower GI tract. 

There remains concern that endoscopic wishbone removal attempts may have contributed to further obstruction, leading to our patient’s subsequent re-presentation with bowel perforation. It is unlikely that microperforation occurred during the case. It is more probable that the inability to remove the foreign body led to subsequent perforation due to pressure necrosis from the wishbone over the two-week outpatient period. This case suggests that patients with foreign bodies impacted in malignant strictures would benefit from definitive surgical management at the time of presentation.

The unusual location of this impacted foreign body in the sigmoid colon was likely due to the patient’s underlying circumferential sigmoid adenocarcinoma. It has been reported that foreign bodies are often found in narrowed or angled parts of GI tracts, such as the ileocecal valve or rectosigmoid junction [8]. Cases of bowel perforation secondary to foreign body presence at alternative GI tract locations have similarly resulted in the discovery of a neoplastic mass, although this is an infrequently reported finding [8,15].

## Conclusions

Thus, we present the case of an ingested foreign body, a wishbone, causing a presenting symptom of abdominal pain without initial colonic perforation. Endoscopic attempts to remove the wishbone were unsuccessful due to an obstructing neoplastic mass, eventually leading to a colonic perforation prior to elective surgical intervention. These unforeseen findings highlight the intricacies and challenges of managing foreign body ingestion. In cases of impacted sharp foreign bodies, it may be advisable to perform definitive surgical management before discharge. Future studies are needed to clarify indications for endoscopic versus surgical management of foreign bodies impacted in malignant strictures in the lower GI tract.
